# Uncovering the molecular mechanisms of lignocellulose digestion in shipworms

**DOI:** 10.1186/s13068-018-1058-3

**Published:** 2018-03-07

**Authors:** Federico Sabbadin, Giovanna Pesante, Luisa Elias, Katrin Besser, Yi Li, Clare Steele-King, Meg Stark, Deborah A. Rathbone, Adam A. Dowle, Rachel Bates, J. Reuben Shipway, Simon M. Cragg, Neil C. Bruce, Simon J. McQueen-Mason

**Affiliations:** 1 Centre for Novel Agricultural Products, Department of Biology, University of York, York, YO10 5DD UK; 2 Bioscience Technology Facility, Department of Biology, University of York, Heslington, York, YO10 5DD UK; 3 Biorenewables Development Centre, 1 Hassacarr Close, Chessingham Park, Dunnington, York, YO19 5SN UK; 4 Marine Science Center, Northeastern University, Nahant, MA 01908 USA; 5 School of Biological Sciences, University of Portsmouth, King Henry Building, King Henry 1st St, Portsmouth, PO1 2DY UK

## Abstract

Lignocellulose forms the structural framework of woody plant biomass and represents the most abundant carbon source in the biosphere. Turnover of woody biomass is a critical component of the global carbon cycle, and the enzymes involved are of increasing industrial importance as industry moves away from fossil fuels to renewable carbon resources. Shipworms are marine bivalve molluscs that digest wood and play a key role in global carbon cycling by processing plant biomass in the oceans. Previous studies suggest that wood digestion in shipworms is dominated by enzymes produced by endosymbiotic bacteria found in the animal’s gills, while little is known about the identity and function of endogenous enzymes produced by shipworms. Using a combination of meta-transcriptomic, proteomic, imaging and biochemical analyses, we reveal a complex digestive system dominated by uncharacterized enzymes that are secreted by a specialized digestive gland and that accumulate in the cecum, where wood digestion occurs. Using a combination of transcriptomics, proteomics, and microscopy, we show that the digestive proteome of the shipworm *Lyrodus pedicellatus* is mostly composed of enzymes produced by the animal itself, with a small but significant contribution from symbiotic bacteria. The digestive proteome is dominated by a novel 300 kDa multi-domain glycoside hydrolase that functions in the hydrolysis of β-1,4-glucans, the most abundant polymers in wood. These studies allow an unprecedented level of insight into an unusual and ecologically important process for wood recycling in the marine environment, and open up new biotechnological opportunities in the mobilization of sugars from lignocellulosic biomass.

**Electronic supplementary material:**

The online version of this article (10.1186/s13068-018-1058-3) contains supplementary material, which is available to authorized users.

## Background

Large amounts of wood from terrestrial plants enter the marine environment and support complex ecosystems. Tropical mangrove swamps, for example, provide safe nurseries that support fisheries [1] and are among the most productive ecosystems on the planet. It has been shown that around 70% of dead wood in mangroves is processed by the action of wood-boring bivalve molluscs known as shipworms [2, 3]. Shipworms acquired their name due to their devastating effects on wooden ships prior to the advent of copper-bottoming, a process developed to protect wooden vessels from their attack. Shipworms continue to destroy timber structures and docks around the world but, despite their ecological, historical and economic importance, the process by which these animals digest wood remains poorly understood.

**Fig. 1:**
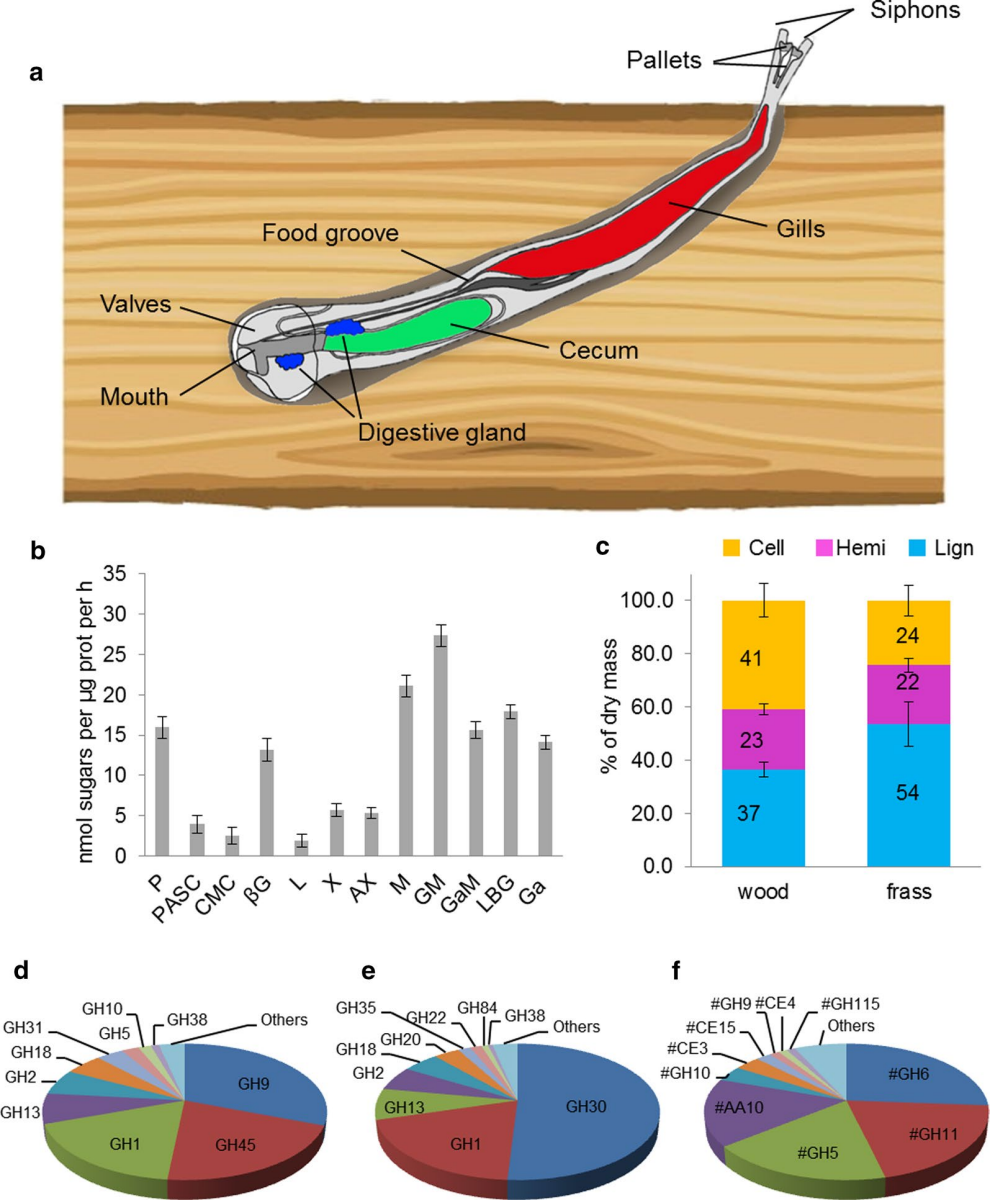
Overview of the anatomy, lignocellulolytic activities and digestive meta-transcriptome of *Lyrodus pedicellatus*. **a** Schematic diagram of *L. pedicellatus*, showing cecum, gills and the two portions (anterior and posterior) of the digestive gland. **b** In vitro activity assay of cecum fluids with a panel of polysaccharides, determined via DNS assay [8]. The detected activities on glucans, mannans and xylans are generally compatible with the putative function of the shipworm GHs based on sequence similarity to characterized proteins. *P* pachyman, *PASC* phosphoric acid swollen cellulose, *CMC* carboxy methyl cellulose, *βG* beta-glucan, *L* lichenan, *X* xylan, *AX* arabinoxylan, *M* mannan, *GM* glucomannan, *GaM* galactomannan, *LBG* locust bean gum, *Ga* galactan. Bars indicate means (error bars: standard deviations of three replicates). **c** Compositional analysis of lignocellulose fractions (*Cell* cellulose, *Hemi* hemicellulose, *Lign* lignin) from Scots pine before (“wood”) and after passing through the shipworm digestive system (“frass”). Bars indicate means (error bars: standard deviations of five replicates). **d**, **e**, **f** Pie charts showing the relative transcript abundance (obtained from TPM values, see “Methods” for more details) of CAZymes identified in digestive gland (**d**), cecum (**e**) and gills (**f**) of *L. pedicellatus*. Enzyme families where all members are of bacterial origin are marked with a pound sign (#)

Shipworms burrow cylindrical tunnels using specialized shell valves with abrasive toothed ridges as a rasp and ingest the wood particles as they burrow. Wood particles are transported by ciliary currents through the esophagus and stomach to accumulate in the cecum [4], which occupies a large part of the animal’s body (Fig. 1a) and is thought to form the main site of wood digestion [2]. While the digestive systems of most herbivorous and xylophagous animals harbor commensal microbes that assist with digestion, the shipworm cecum is reported to be largely devoid of microbial life [5]. However, large amounts of carbohydrate active enzymes (CAZymes) have been shown to be produced by endosymbiotic bacteria housed in specialized cells (bacteriocytes) in the animal’s gills, and have been reported to play a major role in wood digestion by shipworms [6]. The gills are spatially distant from the cecum (Fig. 1a), and the route by which bacterial enzymes move to the site of wood digestion remains elusive. Previous work in *Bankia setacea* suggests that bacterial CAZymes account for the majority of the digestive proteome in shipworms [6]. We have undertaken studies in *Lyrodus pedicellatus* with the aim of better understanding the digestive processes in shipworms, and here reveal the importance of the shipworm’s own enzymes in wood digestion.

## Results

### Meta-transcriptomic analysis of *L. pedicellatus* and its endosymbionts

In vitro activity assays carried out with cecum fluids of *L. pedicellatus* against a panel of substrates revealed a complex enzymatic cocktail with activity against many polysaccharides associated with lignocellulosic biomass (Fig. 1b). Compositional analysis of wood and shipworm feces (frass) revealed that more than 40% of the cellulose content and lesser amounts of hemicellulose are removed while passing through the shipworm digestive system (Fig. 1c). While compelling evidence has shown that gill bacteriocytes are the main site for the production of bacterial CAZymes in shipworms, several authors have also hypothesized that the digestive gland might be responsible for the synthesis of endogenous enzymes, while the cecum appears to be the site of wood breakdown and could potentially be involved in sugar uptake [2, 4]. In order to identify the key genes involved in wood digestion and absorption of breakdown products in *L. pedicellatus*, we performed meta-transcriptome sequencing from the main organs putatively involved in wood digestion (digestive gland, cecum, and gills) (Fig. 1a, Additional file 1: Table S1) using healthy adult *L. pedicellatus* growing in blocks of Scots pine submerged in sea water.

Our gene expression analysis reveals that the shipworm digestive gland is the major site of transcription of endogenous lignocellulolytic enzymes in *L. pedicellatus*, all carrying a predicted signal peptide for secretion (identified using SingalP). BlastX and functional domain annotation show that the most highly transcribed CAZyme genes in the digestive gland encode putative glycoside hydrolases (GHs) belonging to GH9, GH45, GH1, GH13, GH2, GH18, GH31, GH5, GH10, and GH38 families (Fig. 1d, Additional file 1: Table S2).

Very few bacterial transcripts were detected in the cecum samples, confirming previous reports of the virtual absence of live bacteria in this organ [5]. Only two sequences sharing similarity to putative GH30s from *Bacillus* species were found to be expressed at relatively high levels in the cecum (Fig. 1e, Additional file 1: Table S3). However, manual sequence alignment revealed that the two contigs are actually part of one unique transcript featuring a putative polyadenylation (polyA) tail at the 3′ terminus, and have orthologues in the annotated genomes of model bivalve molluscs (XP_011456351.1 from *Crassostrea gigas*, XP_021348230.1 from *Mizuhopecten yessoensis*), whose intron-exon structure strongly suggests an endogenous nature. This GH30 gene might thus be the result of an ancient horizontal gene transfer (HGT) from bacteria, a phenomenon previously observed for several GH families in multiple invertebrate genomes [7]. The shipworm cecum also features high expression of endogenous GH1s, GH13s, and GH2s (Fig. 1e, Additional file 1: Table S3).

RNA-Seq data show that virtually all bacterial genes found in the shipworm meta-transcriptome are transcribed in the gills, with the most abundant being from GH family 6, 11, 5 and auxiliary activity (AA) family 10 lytic polysaccharide monooxygenases (LPMOs) (Fig. 1f, Additional file 1: Table S4). All these bacterial CAZymes carry a putative N-terminal signal peptide likely involved in protein translocation through the periplasm for secretion and have best BlastX matches to sequences from gammaproteobacteria of the family Alteromonadaceae (mainly *Teredinibacter* and *Saccharophagus* species). The identification of the gills as the main site of expression of bacterial CAZymes is in line with previous work from the shipworm *B. setacea*, where GH5s and GH6s were found to be the dominant CAZymes produced by gill bacteria [6].

### Comparison of the digestive gland transcriptome from *L. pedicellatus* and *Crassostrea gigas*

**Fig. 2:**
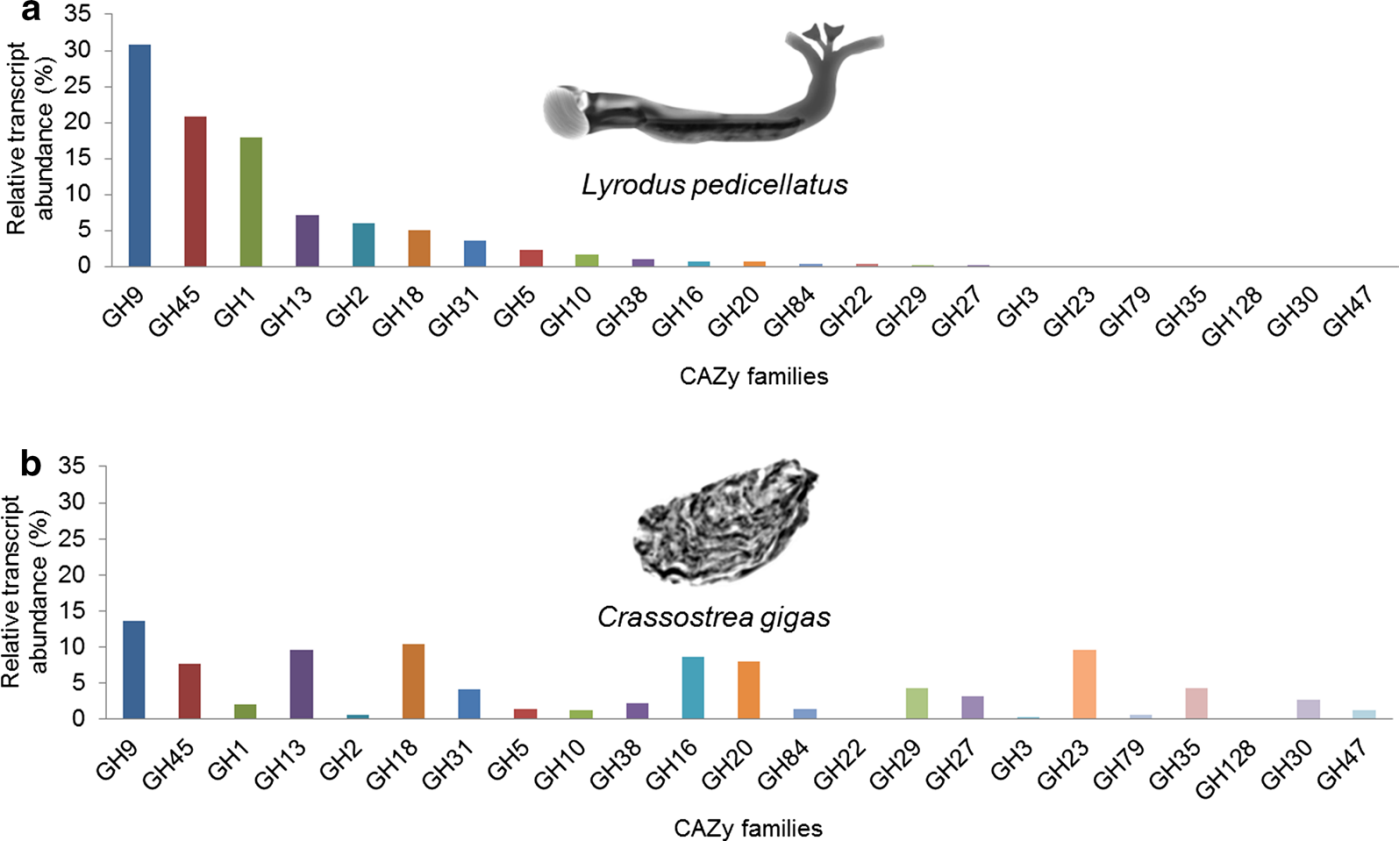
Relative transcript abundance (cumulative) of the endogenous (non-bacterial) glycoside hydrolase families identified in the digestive gland transcriptomes of *L. pedicellatus* (**a**) and *C. gigas* (**b**). Normalized transcript levels were obtained for TPM values (see “Methods” for more details)

*Crassostrea gigas* (Japanese oyster) is a model suspension feeding bivalve with a fully annotated genome and numerous transcriptomic resources readily available through open access databases. We have compared the digestive gland transcriptome of the wood-boring *L. pedicellatus*, with publicly available data from *C. gigas*, in order to try and pinpoint the enzymes that shipworms have uniquely recruited for wood digestion. The results show that, while putative endo β-1,4-glucanases (GH9s and GH45s) and β-glucosidases (GH1s) together account for over 70% of all CAZymes expressed in the digestive gland of the shipworm (Fig. 2a), these classes are much less abundantly expressed in the oyster (Fig. 2b). In contrast, the oyster transcriptome shows a greater abundance of putative endo β-1,3-glucanases (GH16), α-l-fucosidases (GH29), α-galactosidases (GH27), β-galactosidases (GH35), α-mannosidases (GH38, GH47), xylanases (GH30), β-xylosidases (GH3) (Fig. 2b) and aryl-sulfatases (Additional file 1: Figure S1), compatible with the digestion of polysaccharides (such as mannans, laminarin, xylan, sulfated fucans, and galactans) abundant in the cell walls of phytoplankton [9], which represents the staple diet of *C. gigas* [10].

### Proteomic analysis of the shipworm cecum content

**Fig. 3:**
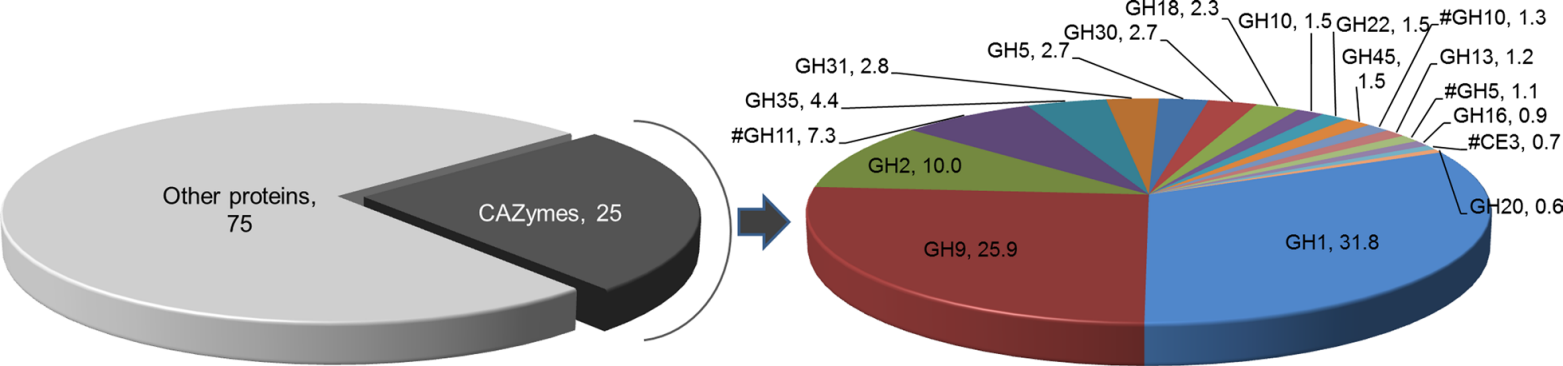
Pie charts showing relative abundance of the CAZy families identified in the proteomic analysis of the cecum content of *L. pedicellatus*. Enzyme families where all members are of bacterial origin are marked with a pound sign (#). Numbers indicate the percentage of molar abundance derived from cumulative emPAI values

Previous work on *B. setacea* concluded that most lignocellulolytic enzymes in the shipworm digestive system were of bacterial origin [6]. The authors, however, did not take into account the contribution of endogenous enzymes by the animal itself. By carrying out shotgun proteomics on the total protein extract from the cecum content, we found that CAZymes represent 25% of the total cecum proteome (Fig. 3), while the remaining 75% includes abundant proteases, immunity-related and structural proteins (data not shown). Our analysis shows that less than 15% of the CAZymes detected in the cecum of *L. pedicellatus* are bacterial, while over 85% are endogenously produced by the animal (Fig. 3). Interestingly, abundant GHs identified in the cecum proteome usually correspond to the most highly transcribed genes in the digestive gland (Fig. 1d), suggesting that the mature proteins are secreted and transported by ciliary tracts to the cecum. GH1 represents the dominant enzyme family in the cecum proteome, and mostly occurs as multi-modular proteins, with domains connected by short peptide linkers. The largest multi-domain GH1 (~300 kDa), identified from both transcriptome and proteome, appears as the predominant band in SDS-PAGE analysis of the crude cecum extract, and its identity was confirmed by tryptic digestion and MALDI-MS/MS analysis (Additional file 1: Figure S2). This sequence is specifically expressed in the digestive gland and bears similarity to lactase phlorizin hydrolase (LPH), an enzyme that is localized at the intestinal brush border membrane in mammals. LPH comprises four distinct GH1 domains and mainly exhibits lactase activity [11].

Interestingly, although GH9s and GH1s are abundant in both transcriptome and proteome, the relative abundance of GH45s is high in the transcriptomic data but lower in the proteome, where it accounts for only 1.5% in molar percentage of total CAZymes identified. The major bacterial contributions to the proteome are provided by GH11, 10 and 5 proteins, which typically function as xylanases, mannanases and endo-glucanases. In *B. setacea*, bacterial GH5s and GH6 were reported to account for over 30% of the total protein content of the cecum [6], but here only make up 0.5% of the total proteome (less than 2% of all CAZymes).

### Isolation and characterization of the multi-domain GH1 from *L. pedicellatus* (*Lp*MDGH1)

**Fig. 4:**
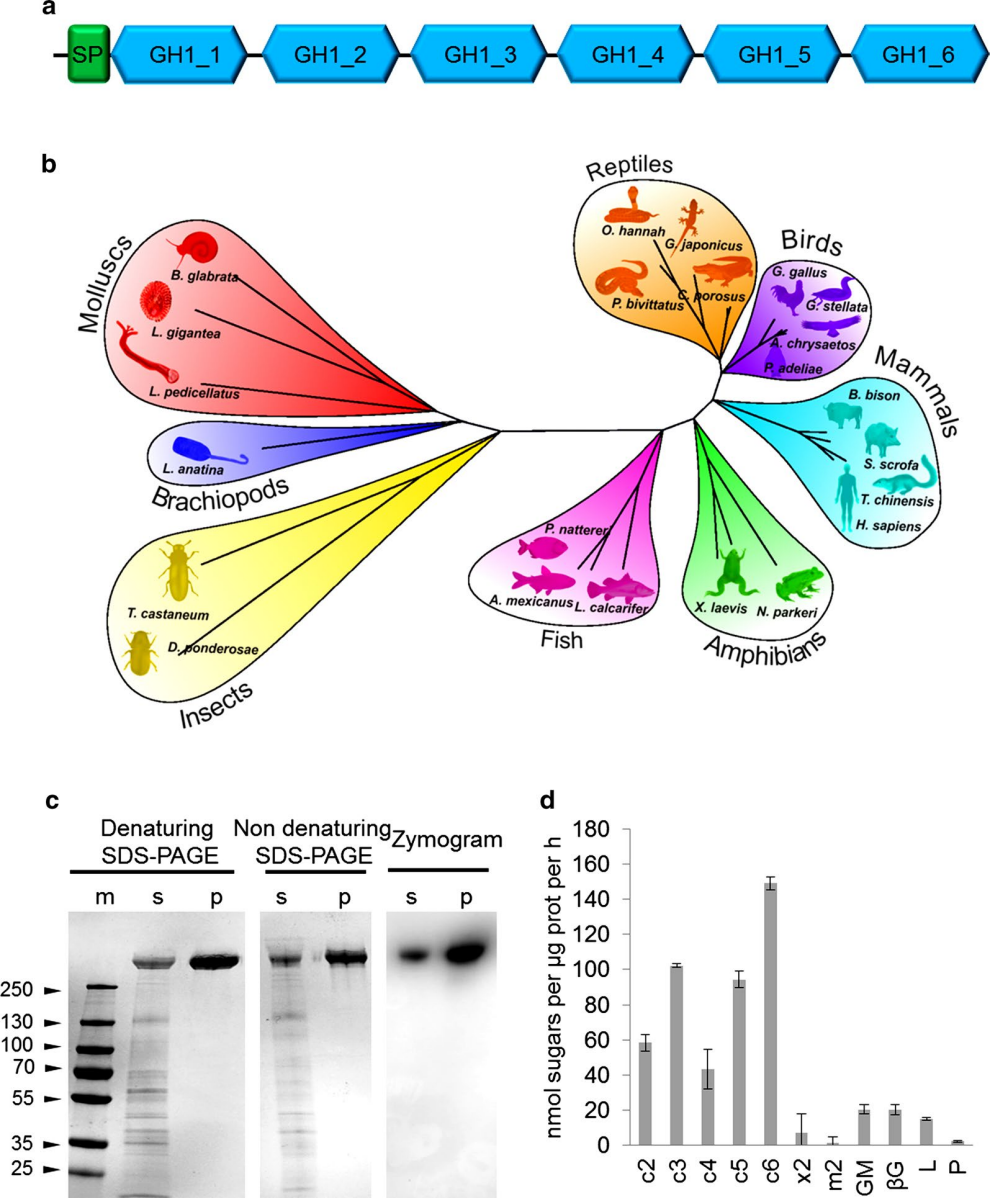
Characterization of *Lp*MDGH1. **a** Schematic diagram of the architecture of the multi-domain GH1 from *L. pedicellatus* (*Lp*MDGH1), featuring an N-terminal signal peptide for secretion and six distinct GH1 domains (numbered from 1 to 6) connected by short peptide linkers. **b** Maximum likelihood radial phylogeny of a subset of multi-domain GH1 proteins identified by BlastP search versus NCBI nr databases. **c** SDS-PAGE (denaturing and non-denaturing) and zymogram of soluble cecum fluids (s) and purified *L. pedicellatus Lp*MDGH1 (p) using the chromogenic substrate 5-bromo-4-chloro-3-indolyl-β-d-cellobioside. A commercial protein marker (m) has been run in the same gel, with numbers representing the molecular weight of the protein bands in kDa. **d** Histogram showing the nanomoles of reducing sugars (as determined via DNS assay) released by the purified *Lp*MDGH1 from cellobiose (c2), cellotriose (c3), cellotetraose (c4) cellopentaose (c5), cellohexaose (c6), xylobiose (×2), mannobiose (m2), konjac glucomannan (GM), barley β-glucan (βG), lichenan (L) and pachyman (P). Activity assays with insoluble polysaccharides included 1 mg mL^−1^ substrate. Activity assays with soluble oligosaccharides included 2.5 mM substrate. Bars indicate means (error bars: standard deviations of three replicates)

Our combined transcriptomic and proteomic analyses show that a novel multi-domain GH1 (*Lp*MDGH1) is among the most highly expressed sequences in the digestive gland and represents the most abundant CAZyme in the shipworm digestive system (over 3% by molar abundance and 20% by mass) and likely plays an important role in wood digestion. In order to verify that the identified sequence is not an artifact of the de novo transcriptome assembly, we cloned the full-length cDNA of *Lp*MDGH1 and confirmed that it comprises a single open reading frame coding for a polypeptide of 2752 amino acids (Additional file 1: Text S1). While the mammalian lactase phlorizin hydrolase (LPH) has four GH1 domains in the immature protein followed by a transmembrane sequence [11], the shipworm gene encodes an N-terminal signal peptide, followed by six GH1 domains and no transmembrane sequence (Fig. 4a), confirming our proteomic observations of a soluble extracellular protein. In mammals, the first half of the LPH protein has been shown to act as a chaperone that facilitates the folding of the second half [12]. The mammalian LPH undergoes several post-translational modifications, and only two domains (3 and 4) are found in the mature protein. In contrast, the shipworm MDGH1 mature protein, found in the cecum, retains all six GH1 domains, as confirmed by MALDI-MS/MS analysis (Additional file 1: Figure S2) and size on SDS-PAGE gels (Additional file 1: Figure S2).

A modular protein (*Cj*Cel1A) comprising 2 sequential GH1s, reminiscent of the mammalian LPH, was shown to be produced in the digestive gland of the clam *Corbicula japonica* [13]. Based on amino acid sequence, it was hypothesized that the anterior part (first GH1 domain) of the protein from *Corbicula* is not active as a glycoside hydrolase and might instead work as a chaperone, in a similar fashion to the mammalian LPH [12]. Alignment of the six putative GH1 modules of the *L. pedicellatus* protein reveals that domains 2, 4, 5 and 6 possess the required amino acids for hydrolytic activity (regions “NE” and “TENG” in the protein alignment), while domains 1 and 3 lack these residues, are unlikely to have GH activity, and might thus be involved in protein folding or perhaps substrate interactions (Additional file 1: Figure S3).

A BlastP search of the full-length *Lp*MDGH1 against non-redundant (nr) databases finds best matches among molluscs (e.g., *Lottia gigantea*) and reveals that LPH-like sequences are also present in insects, reptiles, birds, amphibians, mammals and fish (Fig. 4b), but not in bacteria, fungi and some animal taxa (e.g., crustaceans). The multi-domain GH1s from vertebrates typically feature a putative C-terminal transmembrane region (Additional file 1: Figure S4), likely involved in anchoring the mature protein to the outer face of the cell plasma membrane. Although our analysis indicates that the lack of this transmembrane region is a common trait among multi-domain GH1s from invertebrates (Additional file 1: Figure S4), the six-domain architecture appears unique to the shipworm protein and might, therefore, represent a specific adaptation towards wood digestion.

Despite our best efforts, we could not obtain soluble *Lp*MDGH1 (nor any of its GH1 domains separately) in the heterologous expression systems we tested. However, we carried out size-exclusion chromatography of soluble cecum extracts and successfully isolated the mature *Lp*MDGH1 to high purity. Zymograms and in vitro assays with the purified enzyme showed that it is the major β-glucosidase in *L. pedicellatus* cecum (Fig. 4c). Interestingly, the enzyme showed activity towards both short-chain gluco-oligosaccharides and long-chain glucans (glucomannan, β-glucan, and lichenan), with preference towards β-1,4 linkages, suggesting roles in the digestion of cellulose and hemicelluloses (Fig. 4d). Such a release of sugars from complex glucans was not seen in assays with a commercially sourced single-module GH1 from *Agrobacteri*a (data not shown) and might be an unusual feature of the multi-modular protein. MALDI-TOF MS analysis of the reaction products revealed that *Lp*MDGH1 releases medium- and long-chain oligosaccharides from glucans, suggesting that this enzyme might also have endo-glucanase activity (Additional file 1: Figure S5). Interestingly, a soluble 210 kDa enzyme isolated from the digestive fluids of the sea hare *Aplysia kurodai* was shown to share high similarity with the human lactase phlorizin hydrolase (based on N-terminal protein sequencing) [14]. Although the authors did not manage to isolate the coding sequence nor localize the organ where it was produced, they showed that the purified enzyme can hydrolyze gluco-oligosaccharides as well as complex polysaccharides (lichenan, laminarin, and cardran), thus suggesting a key role in the digestion of sea lettuce by the sea hare [14]. The protein size, in vitro activity, and sequence similarity to mammalian LPH suggest that the enzyme from the sea hare has four GH1 domains and plays a similar role to the MDGH1 from *L. pedicellatus*.

### Anatomy of the digestive system

**Fig. 5:**
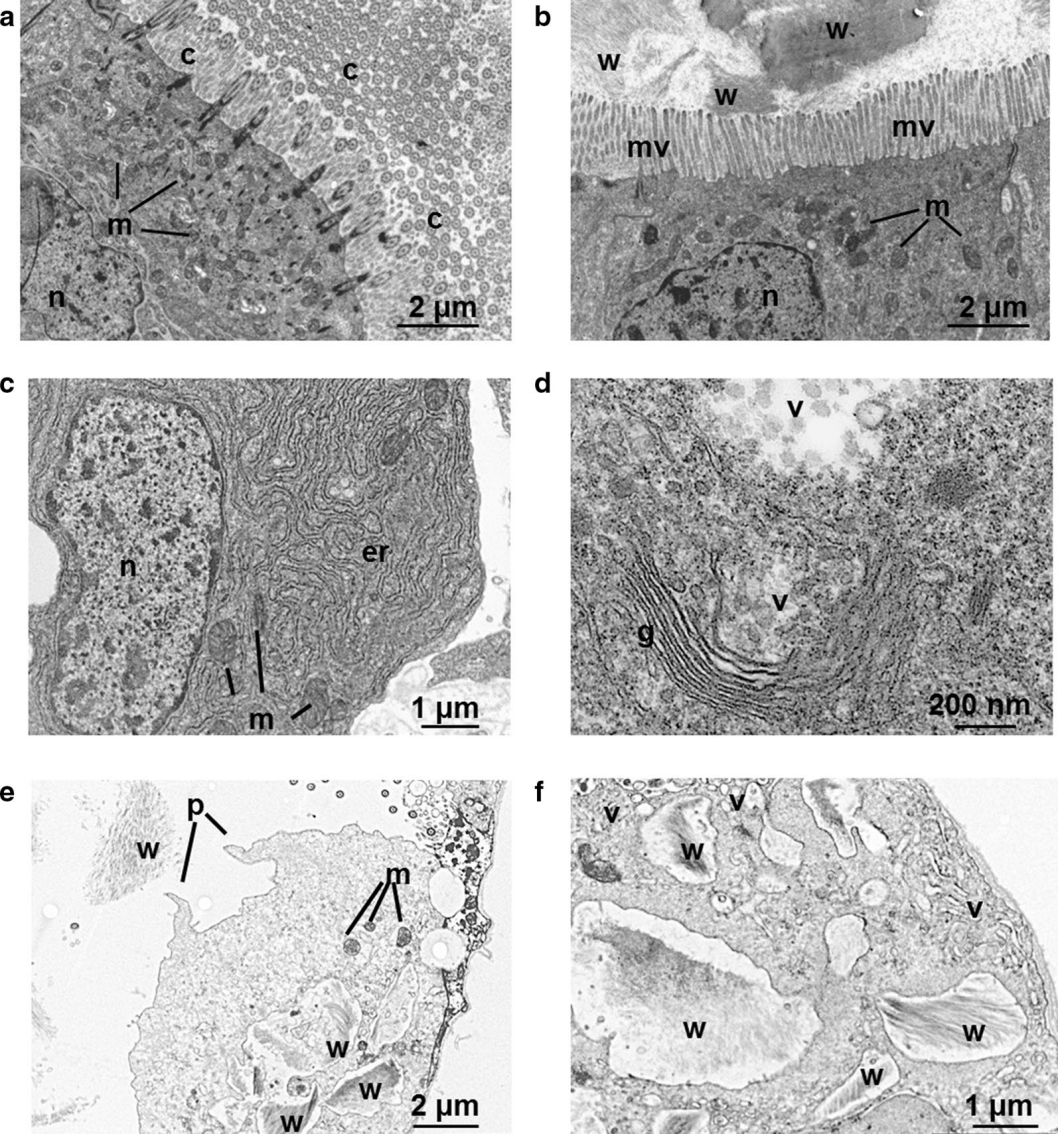
Transmission electron microscopy (TEM) of the cecum and digestive gland from *L. pedicellatus*. **a** Cecum showing abundant cilia (c) projecting from the apical surface of the cell. **b** Cecum epithelium. **c** Digestive gland secretory cell with electron-dense endoplasmic reticulum. **d** Golgi apparatus and putative secretory vesicles. **e** Digestive gland phagocyte, showing internalized wood particles and pseudopodia. **f** Wood-engulfing vesicles in a gland phagocyte. *c* cilia, *er* endoplasmic reticulum, *g* Golgi apparatus, *mv* microvilli, *n* cell nucleus, *m* mitochondrion, *p* pseudopodium, *v* vesicle, *w* wood particle

Visible light and electron microscopy analysis of sections of the shipworm’s digestive system show that wood particles coming from the grinding action of the valves accumulate in the cecum, where most lignocellulose breakdown is thought to occur [2, 5] . Examination of the cecum luminal walls via electron microscopy revealed high abundance of microvilli and cilia at the apical surface of the cells (Fig. 5a, b), confirming the previously hypothesized role of the cecum in agitating food particles and in the absorption of breakdown products (sugars) from wood digestion [2]. This absorptive function is supported by the abundant expression of putative glucose transporters (solute carrier family 2 transporters and sodium-dependent glucose transporters) in the cecum tissues (Additional file 1: Figure S6).

Although the cecum contains most of the ingested wood particles, the digestive gland has long been hypothesized to be involved in production of digestive enzymes in shipworms and other molluscs [15–22], and our meta-transcriptomic and proteomics data confirm this is the case for *Lyrodus*. The gland has a lobular structure reminiscent of secretory organs in other animals and is directly connected to the stomach by ducts [4, 22]. Our high-resolution microscopic analyses reveal that the gland contains secretory cells, as previous observed in other molluscs [19–21]. These specialized cells occupy the crypts of the tubule, are mostly pyramidal, and have a well-developed granular endoplasmic reticulum (Fig. 5c) and an extensive Golgi apparatus, producing numerous vesicle-like structures (Fig. 5d) of variable sizes potentially containing glycoside hydrolases and other secretory enzymes identified in our studies. The digestive gland also features abundant amoeboid cells (phagocytes, ~20 μm in diameter) with pseudopodia and internalized wood particles of variable dimensions (Fig. 5e, f). EM images show numerous vesicles budding from the Golgi apparatus and apparently fusing with the cell wall of the cavities containing the internalized wood fragments (Fig. 5f), suggesting the formation of lysosomes that might be responsible for intracellular wood breakdown. Although wood phagocytes have been previously reported in shipworms with hand drawings [22], this is the first high-resolution image of these cells and provides new evidence of their role in lignocellulose digestion. This suggests that, while the cecum provides the major site of wood digestion, it may be supplemented by intracellular digestion. Indeed, the cecum appears to be a specific adaptation of shipworms and has not been reported in other bivalves, where most digestive processes are restricted to the gland and intestinal systems.

## Discussion

Lignocellulose represents the most abundant and ubiquitous organic material in nature. The breakdown of this recalcitrant polymer plays a critical role in the global carbon cycle, and is attracting growing interest from a biotechnological perspective. As society moves away from the use of net greenhouse gas-emitting fossil resources, the use of surplus woody biomass to provision fuels, chemicals, and materials is becoming imperative. Understanding the digestive systems of major wood-digesting animals provides insights and enzymes that could help towards the cost-effective breakdown of lignocellulosic biomass into simple sugars and other building blocks. We have undertaken a detailed and multifaceted study of the digestive system of *L. pedicellatus*, and revealed the complex molecular mechanisms of lignocellulose digestion in shipworms. Our work shows that the digestive gland of *L. pedicellatus* produces a complex enzymatic mixture containing most of the activities required for the digestion of the plant cell wall, including cellulases (endo-β-1,4-glucanases, β-glucosidases) and hemicellulases (β-mannanases/mannosidases, β-xylanases/xylosidases). Although the meta-transcriptome of *L. pedicellatus* lacks endogenous cellobiohydrolases (CBHs), it contains bacterial GH6s (which can act as CBHs) expressed in the gills, as previously observed in *B. setacea* [6]. Similarly, we also detected expression of bacterial lytic polysaccharide monooxygenases (LPMOs), which likely synergize endogenous as well as bacterial glucanases. Indeed, previous work has shown that LPMOs can insert breaks into highly crystalline polysaccharides and, by doing so, boost the activity of glycoside hydrolases by several orders of magnitude [23–25]. The surprisingly low levels of both GH6 and LPMO mature proteins in the shipworm cecum, however, suggest that the corresponding transcripts might not be translated efficiently, or that the mature enzymes are unstable in the shipworm digestive tract. In support of this, our cecum proteomics analysis revealed abundant endogenous proteases, which might reduce the half-life of some bacterial enzymes.

Our work in *L. pedicellatus*, and previous studies on wood-feeding cockroaches, beetles, and termites [26–28], suggest that a combination of endogenous and symbiotic enzymes is optimal for efficient plant cell wall digestion in invertebrates. Interestingly, the genomes of insects, crustaceans, annelids and molluscs encode numerous enzymes involved in plant cell wall digestion, implying that some of these genes were present in the last common ancestor of bilaterian animals [29], before bacterial symbioses developed, and likely represent an ancestral mechanism for lignocellulose digestion. Our analysis of the digestive gland transcriptome from shipworm and oyster confirms that molluscs share a complex array of endogenous lignocellulolytic enzymes, and that their expression levels are adapted to their specific diets. *C. gigas* (oyster) is characterized by high expression of glycoside hydrolases and sulfatases involved in the deconstruction of sulfated polysaccharides that are abundant in marine algae (as an adaptation to highly ionic environment) [30] but not in fresh water algae and terrestrial plants. Our data show that *L. pedicellatus* relies mostly on GH9s, GH45s, and GH1s to breakdown terrestrial woody plants, and the same is likely true for most shipworm species, which typically feed on submerged wood. There are, however, some notable exceptions. For example, *Zachsia zenkewitschi* feeds on the rhizomes of seagrasses such as *Zostera*, one of the few examples of marine angiosperms to have regained the ability to produce sulfated polysaccharides [31]. Even more puzzling is the giant mud-boring teredinid *Kuphus polythalamia*, where sulfur-oxidizing bacteria have replaced the ancestral cellulolytic symbionts in the gills [32]. Further work is needed to elucidate the function of the digestive gland in this enigmatic chemoautotrophic bivalve, which represents a unique example of a shipworm that has entirely lost the ability to digest plant biomass.

Previous studies in the shipworm *B. setacea* suggested that glycoside hydrolases produced by endosymbiotic bacteria located in the gills dominate the shipworm digestive system [6]. The apparent discrepancies in results between our study and that by O’Connor et al. [6] may be in part due to differences between species. Both belong to the family Teredinidae, rely on a diet of wood, and share some key anatomical features, including digestive glands, extended cecum, and the presence of analogous bacterial populations in the gill bacteriocytes (mostly gammaproteobacteria related to *Saccharophagus degradans*). Therefore, it would be of interest to determine what factors underpin the differences between our results and those reported by O’Connor et al. [6]. It is worth noting that in the study by O’Connor et al. [6] the cecum proteomic data were reported to be searched specifically against bacterial DNA extracted from the gills, which would have precluded identification of proteins produced by the animal itself. Indeed, our proteomics studies reveal that the bulk of the CAZymes found in the cecum of *L. pedicellatus* is secreted by a specialized digestive gland, while bacterial enzymes coming from the gills play a supporting role. The size of the cecum and abundance of GHs found there suggest that this is the major site of wood digestion in shipworms. Yet, our ultrastructural studies indicate a potential role in wood digestion for ameboid cells in the digestive gland. Although intracellular wood digestion is unusual, it has also been inferred from microscopic analysis of commensal ciliates found in the digestive system of lower termites, which appear to engulf wood particles [33].

By investigating the cecum meta-proteome, we have discovered that the most abundant enzyme in the *L. pedicellatus* digestive tract is an unusual multi-modular GH1 with sequence similarity to the lactase phlorizin hydrolase found in mammals. In contrast to the mature peptide in the mammalian LPH, which has two GH1 domains linked by a short peptide and is mostly active as a β-glucosidase, the *L. pedicellatus* MDGH1 has six domains, and our studies have revealed its specificity against β-linked glucosides as well as complex glucans, suggesting the ability to cleave these linkages in cellulose and glucomannans during wood digestion. Based on the presence of key amino acid residues predicted to be involved in substrate attack, we would expect four of the six GH1 domains in the *Lp*MDGH1 to be active glycoside hydrolases. Future work is needed to clarify if the endo- and exo-activities observed in *Lp*MDGH1 depend on the distinct GH1 domains or rather on the fusion nature of the polyprotein, and whether the two putatively inactive GH1 domains play any role in the two mechanisms of action.

The sustainability of biorefineries hinges on the identification of the most effective enzymatic cocktails for the saccharification of plant biomass. Our investigation into the digestive system of *L. pedicellatus* uncovers a wide range of new glycoside hydrolases attacking major fractions of lignocellulose (cellulose, xylans, and mannans) and unprecedented information regarding their relative abundance, which could help engineer an optimal enzymatic cocktail for the breakdown of lignocellulose. In this context, the identification of *Lp*MDGH1 as the major enzyme in the shipworm’s cecum is particularly interesting, as one of the major bottlenecks in the industrial breakdown of plant biomass is the ability to prevent the accumulation of cellobiose, a potent inhibitor of endo-glucanases and cellobiohydrolases [34]. Shipworms seem to have overcome this issue by mass producing a unique multi-domain hydrolase with dual activity towards long-chain glucans and cellobiose, an elegant evolutionary solution that may help simplify the enzymatic cocktails used in cellulosic biorefineries.

## Conclusions

This work is the first comprehensive investigation of the complex molecular and physiological processes by which shipworms extract nutrients from wood and play a fundamental role in the global carbon recycling. The identification of the key enzymes produced by the shipworm *L. pedicellatus*, and the in vitro characterization of the most abundant glycoside hydrolase found in its digestive system, may open up new opportunities in the biotechnological deconstruction of lignocellulose in support of a sustainable bio-economy.

## Methods

### Substrates

Phosphoric acid swollen cellulose (PASC) was prepared as follows. 5 g of Avicel^**®**^ PH-101 was moistened with water and treated with 150 mL ice cold 85% phosphoric acid, stirred on an ice bath for 1 h. Then 500 mL cold acetone was added while stirring. The swollen cellulose was filtered on a glass-filter funnel and washed 3 times with 100 mL ice cold acetone and subsequently twice with 500 mL water. PASC was then suspended in 500 mL water and blended to homogeneity.

High-purity pachyman (β-d-1,3-glucan), barley β-glucan (β-d-1,3-1,4-glucan), lichenan (from Icelandic moss, β-d-1,3-1,4-glucan), mannan (borohydride reduced), konjac glucomannan (β-d-1,4), carob galactomannan, larch arabinogalactan, wheat arabinoxylan, cellotriose, cellotetraose, cellopentaose, cellohexaose, mannobiose, and xylobiose were purchased from Megazyme. Locust bean gum, carboxymethyl cellulose (CMC), beechwood xylan, and cellobiose were purchased from Sigma. 5-bromo-4-chloro-3-indolyl-β-d-cellobioside was purchased from Santa Cruz Biotechnology.

### Specimen collection

Adult *Lyrodus pedicellatus*, matching the sequences of cytochrome c oxidase subunit I and small subunit rRNA 18S of the Atlantic population of *Lyrodus* as per Borges et al. [35], were obtained from an infested pier composed of greenheart wood (*Chlorocardium rodiei*) at Portsmouth, UK. Adults were harvested and dissected in order to yield the larvae. Subsequent cultures were reared in aquaria at the Institute of Marine Sciences, University of Portsmouth. Seawater was taken directly from Langstone Harbour, maintained at a temperature between 15–18 °C and a salinity of 33 PSU, and kept aerated throughout. Tanks were regularly provided with small panels of Scot pine wood for larval settlement.

### mRNA extraction, preparation, and sequencing

Digestive gland, cecum and gills were dissected from three healthy adult *L. pedicellatus* and total RNA was extracted using TRIzol^®^ Reagent (Thermo Fisher Scientific). Samples were DNase treated using Turbo DNA-free (Ambion) before quantification using a Qubit Fluorometer (Thermo Fisher Scientific). Ribosomal RNA depletion was carried out with a RiboZero™ Magnetic Gold Kit (Epidemiology) (Epicentre). mRNA was then concentrated using RNA Clean & Concentrator™-5 (Zymo Research). RNA-Seq libraries were prepared from each mRNA sample as per the Ion Total RNA-Seq kit v2 (Thermo Fisher Scientific), using an RNaseIII treatment time of 2.5–3 min. Samples were barcoded using the Ion Xpress RNA-Seq Barcode kit (Thermo Fisher Scientific). Yields and library sizes were assessed using the High Sensitivity D1K screentapes and reagents on a 2200 TapeStation Nucleic Acids System (Agilent Technologies). Appropriately diluted library aliquots were combined in pairs in equimolar amounts and used for template preparation using the Ion OneTouch 200 Template Kit v2 DL on a OneTouch system (Thermo Fisher Scientific) prior to loading onto a 318 chip and sequenced on an Ion Torrent PGM™ prepared as per the manufacturer’s instructions (IonPGM200Kit; Thermo Fisher Scientific). All raw sequence data were deposited in NCBI under BioProject PRJNA412369 (SRA files: SRR6106265, SRR6106266, SRR6106267, SRR6106268, SRR6106269, SRR6106270, SRR6106271, SRR6106272, SRR6106273). Assembled contigs are available from the authors upon request.

### Transcriptome assembly, sequence annotation, and identification of putative CAZymes

After removing the primer sequences and low-quality reads from raw EST sequencing reads, the EST sequences from three tissues (digestive gland, cecum, and gills) from three healthy adults were assembled into unigene contigs using Trinity [36]. The contigs from the three animals were then assembled into supercontigs with the CAP3 DNA Sequence Assembly Program [37]. Raw reads were mapped onto the transcriptomes of the three molluscs and normalized expression values (TPM = Transcripts per kilobase Million) were calculated for each transcript using Salmon (part of the Galaxy toolshed) [38]. Although all three animals provided raw reads with high quality and could be used to assemble a reference transcriptome, TPM values from one animal were found to be poorly correlated with the other two (possibly as a result of illness or distress) and were therefore excluded from the following analysis. Average TPM values for each contig in the three tissues (digestive gland, cecum, and gills) were thus calculated from the two healthy animals. The top 10,000 assembled contigs (ranked according to the cumulative number of mapped ESTs from all organs) were annotated with the BLASTx algorithm [39] to search against non-redundant (nr) peptide database downloaded from the National Center for Biotechnology Information (http://www.ncbi.nlm.nih.gov/). CAZy annotation was carried out using the CAZYmes Analysis Toolkit (CAT) on the BioEnergy Science Center website (http://mothra.ornl.gov/cgi-bin/cat/cat.cgi). Sequences annotated as glycosyltransferases (GTs) and carboxyl esterases, mostly involved in intracellular processes not relevant to lignocellulose digestion, were excluded from the analysis. Among auxiliary activity (AA) families, only those clearly involved in polysaccharide degradation were considered (LPMO families AA9, AA10, AA11, and AA13).

Contigs were converted to putative ORFs using the online tool Emboss (http://www.bioinformatics.nl/cgi-bin/emboss/getorf), putative N-terminal signal peptides were predicted with SingalP (http://www.cbs.dtu.dk/services/SignalP/), and putative transmembrane regions were predicted using TMHMM (http://www.cbs.dtu.dk/services/TMHMM/).

Raw transcriptome sequencing data for the digestive gland of a wildly caught *C. gigas* (Japanese oyster) were retrieved from the EBI portal (run Accession SRR334213) [40]. The published transcriptome of *C. gigas* (based on the annotated genome) was retrieved from NCBI (Accession PRJNA276446). Raw reads were mapped onto the transcriptome of the mollusc and normalized expression values were calculated for each transcript using Salmon (part of the Galaxy toolshed) [38]. The identity and relative abundance of the CAZyme families were then compared to those obtained from the digestive gland of *L. pedicellatus*. CAZy annotation was carried out using the CAZYmes Analysis Toolkit (CAT) on the BioEnergy Science Center website (http://mothra.ornl.gov/cgi-bin/cat/cat.cgi).

### Proteomics analysis

The ceca from five animals grown on Scots pine were dissected in 50 mM sodium phosphate buffer pH 7 and the content (food particles and enzymes) was collected, pooled together, added with 1% SDS, beta-mercapto ethanol, DTT, boiled for 10 min, centrifuged, and the supernatant run into a 10% polyacrylamide gel to a depth of 1 cm, before staining with Coomassie.

In-gel tryptic digestion was performed post reduction with DTE and *S*-carbamidomethylation with iodoacetamide. Resulting peptides were analyzed by label-free LC-MS/MS over a 125-min gradient using a Waters nanoAcquity UPLC interfaced to a Bruker maXis HD mass spectrometer as detailed in [41]. Protein identification was performed by searching tandem mass spectra against the assembled transcriptome of *L. pedicellatus* using the Mascot search program. Matches were passed through Mascot percolator to achieve a false discovery rate of < 1% and further filtered to accept only peptides with expect scores of 0.05 or better. Molar percentages were calculated from Mascot emPAI values by expressing individual values as a percentage of the sum of all emPAI values in the sample [42]. Proteins identified in the proteomics analysis were annotated via Blastx versus non-redundant NCBI databases. CAZy annotation was carried out using the CAZYmes Analysis Toolkit (CAT) on the BioEnergy Science Center website (http://mothra.ornl.gov/cgi-bin/cat/cat.cgi).

One aliquot of the cecum extract (content only) and the purified *Lp*MDGH1 was added with 1% SDS, beta-mercapto ethanol, DTT, boiled for 10 min, centrifuged, and the supernatant run in a 4–20% gradient polyacrylamide gel. After Coomassie staining, the most abundant band (with an approximate molecular weight of 300 kDa) was excised and in-gel digested as described for LC-MS/MS samples.

A 1 μL aliquot of peptide mixture was applied directly to a ground steel MALDI target plate and overlaid with an equal volume of a 5 mg/mL 4-hydroxy-α-cyano-cinnamic acid in 50% aqueous (v:v) acetonitrile containing 0.1% trifluoroacetic acid (v:v). Positive-ion MALDI mass spectra were obtained using a Bruker Ultraflex III in reflectron mode, equipped with a Nd:YAG smart beam laser. MS spectra were acquired over a range of *m*/*z* 800–4000. The ten most intense precursors with *S*/*N* greater than 30 were selected for MS/MS fragmentation in LIFT mode without collision gas. The default calibration was used for MS/MS spectra, which were baseline-subtracted and smoothed (Savitsky–Golay, width 0.15 *m*/*z*, cycles 4); monoisotopic peak detection used a SNAP averaging algorithm (C 4.9384, N 1.3577, O 1.4773, S 0.0417, H 7.7583) with a minimum *S*/*N* of 6. Bruker flexAnalysis software (version 3.3) was used for spectral processing and peak list generation. Tandem mass spectral data were submitted to database searching using a locally running copy of the Mascot program (Matrix Science Ltd., version 2.5), through the Bruker ProteinScape interface (version 2.1). Search criteria included enzyme, trypsin; fixed modifications, carbamidomethyl (C); variable modifications, oxidation (M), deamidated (N, Q); peptide tolerance, 100 ppm; MS/MS tolerance, 0.5 Da; Instrument, MALDI-TOF–TOF. Peptide matches were filtered to require expect scores of 0.05 or better.

### Cloning the *Lp*MDGH1 cDNA

The native sequence (from start to stop codon) for *Lp*MDGH1 was cloned from cDNA generated from RNA extracted from the digestive gland of *L. pedicellatus* using external oligonucleotide primers designed on the assembled contig from the transcriptome. Total RNA was extracted from one animal using the TRIzol^**®**^ Reagent (Thermo Fisher Scientific) and cDNA was generated with an oligodT primer using SuperScript^**®**^ II reverse transcriptase (Thermo Fisher Scientific). PCR reactions were then set up using Phusion^®^ High-Fidelity DNA Polymerase (Thermo Fisher Scientific) and the amplicon was cloned into an auxiliary plasmid using the StrataClone Blunt PCR Cloning Kit (Stratagene) and the correct sequence was verified with the Sanger method using internal primers. Open reading frames (ORFs) were calculated using the online EXPASY tool “Translate” and confirmed that the cloned sequence codes for a unique polypeptide of 2752 amino acid residues (without internal stop codons). The sequence has been deposited in GenBank with Accession No. MG013499.

### Phylogeny and sequence analysis of *Lp*MDGH1

A protein sequence alignment of the single GH1 domains from *Lp*MDGH1 was obtained using T-Coffee [43] and visualized using JalView [44].

The *Lp*MDGH1 protein sequence was searched via BlastP against NCBI non-redundant databases and orthologues from molluscs, insects, fish, amphibians, reptiles, birds, and mammals were retrieved. The resulting amino acid sequences were aligned using Muscle [45], operating with default parameters. A distance matrix was made with Mega6 [46] using a Jones–Taylor–Thornton matrix and a phylogenetic tree was then calculated by the maximum likelihood algorithm and standard parameters. The resulting tree was visualized using Dendroscope [47].

### Purification of *Lp*MDGH1

The ceca of twenty *L. pedicellatus* specimens were dissected in 50 mM sodium phosphate buffer pH 7 and the content (wood particles and enzymes) was collected and centrifuged. The supernatant was then filtered with 0.22-μm syringe filters and applied to a Superose™ 6 Increase 10/300 GL size-exclusion chromatography column (GE Healthcare) pre-equilibrated with 20 mM Tris-HCl pH 8 plus 100 mM NaCl. Eluted fractions were analyzed by denaturing SDS-PAGE and those corresponding to the *Lp*MDGH1 were pooled, concentrated using Microsep™ Advance Centrifugal Filters (Pall Laboratory, 100 kDa cut-off), and re-applied to the same column pre-equilibrated with 20 mM sodium phosphate buffer pH 7 plus 100 mM NaCl. Protein purity was again assessed via SDS-PAGE analysis.

### Enzymatic assays and zymograms

Activity of the cecum fluids on a panel of polysaccharides and oligosaccharides was determined by DNS reducing sugar assay [8]. Briefly, ten ceca were dissected in 50 mM sodium phosphate buffer pH 7 and the content fully re-suspended by pipetting. After centrifugation, the soluble portion (supernatant) was filtered through 0.22-μm porous membranes, quantified with the Bradford [48] reagent and used for assays. 50 μL reactions were carried out in 96-well plates in 50 mM sodium phosphate buffer pH 6 with either 1187 ng of total soluble cecum protein or 237 ng of purified GH1, and 1 mg mL^−1^ polysaccharide or 2.5 mM oligosaccharide. All reactions, including controls, were performed in triplicate. The micro-plate was incubated at 28 °C shaking at 320 rpm for 24 h, and then 100 μL of DNS reagent was added to each reaction before heating at 100 °C for 5 min. Absorbance at 540 nm was measured with a micro-plate reader and nanomoles of reducing sugars released were determined based on absorbance obtained with glucose standards. The DNS reagent was prepared by mixing 0.75 g of dinitrosalicylic acid, 1.4 g NaOH, 21.6 g sodium potassium tartrate tetrahydrate, 0.53 mL phenol, and 0.59 g sodium metabisulfite in 100 mL pure water.

Zymograms were performed as follows. 1.9 μg of total protein from the cecum fluids and purified *Lp*MDGH1 were run in a non-denaturing SDS-PAGE gel (4–20% Mini-PROTEAN^®^ TGX™ Precast Protein Gel, Biorad). The gel was then incubated in 20 mL of 20 mM sodium phosphate buffer pH 6 plus 2.5% Triton X100 for 30 min, and then washed twice in 20 mL of 20 mM sodium phosphate buffer pH 6 for 10 min. The gel was then incubated for 4 h in 20 mL of 20 mM sodium phosphate buffer pH 6 with 0.01% (w/v) of 5-bromo-4-chloro-3-indolyl-β-d-cellobioside to allow formation of the insoluble dye.

### Compositional analysis of wood and frass

Untreated Scots Pine (powdered) and dried frass (obtained from *L. pedicellatus* grown on submerged panels of Scots Pine) were analyzed for cellulose, hemicellulose, and lignin content in five technical replicates each, using the methods reported in Marriot et al. [49].

### Transmission electron microscopy

Dissected shipworm tissue (cecum, digestive gland) was fixed for 1–2 h at ambient temperature in primary fixative (4% formaldehyde (w/v), 2.5% (w/v) glutaraldehyde in 100 mM sodium phosphate buffer pH 7.2), and then washed (3 × 10 min) in 100 mM sodium phosphate buffer pH 7.2 before incubation in secondary fixative for 1 h on ice (1% osmium tetroxide in 100 mM sodium phosphate buffer pH 7.2). Samples were dehydrated through a graded ethanol series (15 min each), followed by two washes (5 min each) in epoxy propane. Samples were infiltrated with a series of epoxy propane/Epon araldite (25%, 50%, 75% Epon Araldite with a minimum of 1 h at each stage, all at 30°C) concluding with a minimum of two changes of Epon araldite resin over 24 h at 30 °C, and polymerized at 60 °C for 48 h in flat embedding molds. Pale gold (70–90 nm) ultra-thin sections were cut with a Diatome diamond knife, using a Leica Ultracut UCT microtome, and mounted on hexagonal 200-mesh nickel grids. Sections were post-stained with 2% (w/v) aqueous uranyl acetate (10 min), then with lead citrate (5 min) [50] in a carbon dioxide-free chamber and viewed using a FEI Tecnai 12 BioTWIN G2 TEM operating at 120  kV. Images were captured using AnalySIS software and a Megaview III CCD camera.

## Additional file

**Additional file 1.** Additional tables, figures, and additional text.
