# Supplementary material for: Uncovering the molecular mechanisms of lignocellulose digestion in shipworms
Source: Biotechnol Biofuels. 2018 Mar 7;11:59. doi: 10.1186/s13068-018-1058-3 (PMC5840672; doi:10.1186/s13068-018-1058-3)
Supplement: Supplementary file 1 — Additional file 1. Additional tables, figures, and additional text. [file 13068_2018_1058_MOESM1_ESM.docx]

Additional Information

**Uncovering the molecular mechanisms of lignocellulose digestion in shipworms**

**Authors:** Federico Sabbadin^a,1^, Giovanna Pesante^a,1^, Luisa Elias^a^, Katrin Besser^a^, Yi Li^a^, Clare Steele-King^a^, Meg Stark^b^, Deborah A. Rathbone^c^, Adam A. Dowle^b^, Rachel Bates^b^, J. Reuben Shipway^d^, Simon M. Cragg^e^, Neil C. Bruce^a^, Simon J. McQueen-Mason^a, 2^

Fig. S1. Transcript abundance of putative sulfatases identified in the digestive gland transcriptomes of *L. pedicellatus* and *C. gigas*. Pfam annotation was performed with the CAZYmes Analysis Toolkit (CAT) on the BioEnergy Science Center website (<http://mothra.ornl.gov/cgi-bin/cat/cat.cgi>), setting an e-value threshold of 10^-5^. Transcript abundance is based on TPM (Transcript Per kilobase Million) values and is relative to the cumulative TPM of all CAZymes identified in the gland of each animal. In order to increase the stringency of the analysis and avoid artefactual over representation of short (partial) sequences, only contigs/transcripts longer than 500 bp were considered.


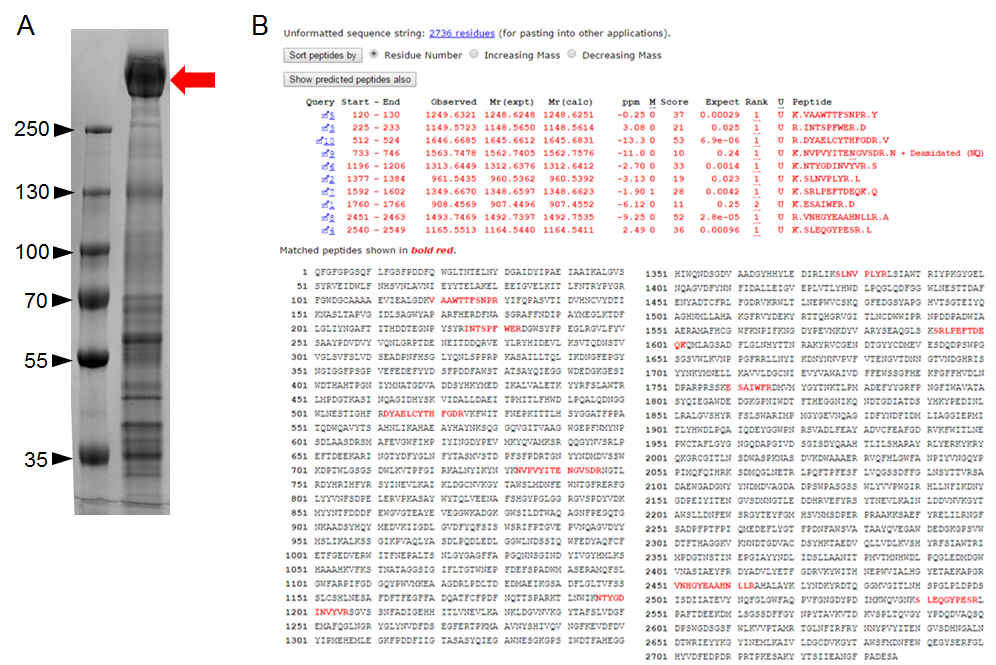


Fig. S2. MALDI-MS/MS analysis of the *Lp*MDGH1 protein band from cecum fluids (A), showing the identification of peptides generate by tryptic digestion (B, original Mascot output format).


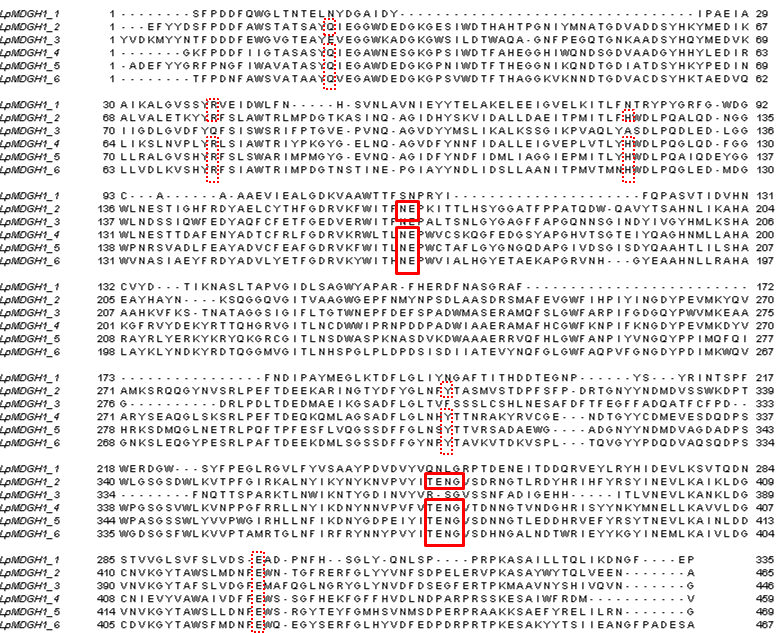


Fig. S3. Protein sequence alignment of the six GH1 domains from *Lp*MDGH1.

Catalytically important motifs and amino acid residues involved in substrate binding that are conserved among GH1s (12) are highlighted with solid and dotted boxes, respectively.


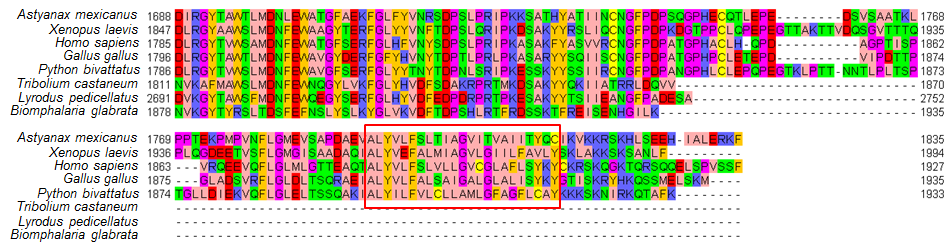


Fig. S4. Protein sequence alignment of the C-terminus from selected multi domain GH1s, showing the putative transmembrane domain.

Protein sequences predicted from genomic data were retrieved from NCBI and aligned using Muscle. Targets were chosen from *A. mexicanus* (fish, accession XP_015463113.1), *X. laevis* (amphibian, accession XP_018092335.1), *H. sapiens* (mammal, accession AAA59504.1), *G. gallus* (bird, accession NP_001104816.1), *P. bivattatus* (reptile, accession XP_015745097.1), *T. castaneum* (insect, accession KYB26300.1) and *B. glabrata* (mollusc, accession XP_013084449.1). The full protein sequence of *Lp*MDGH1 (from *L. pedicellatus*, mollusc) was also included in the alignment. Analysis of the sequences with the tool TMHMM (<http://www.cbs.dtu.dk/services/TMHMM/>) identified a putative transmembrane helix only among vertebrates (red box).


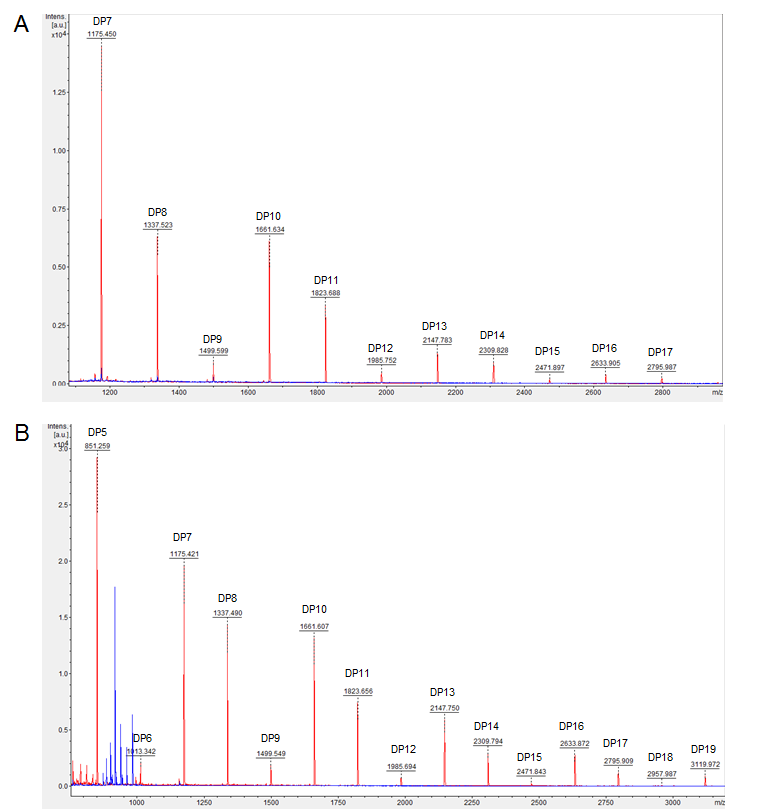


Fig. S5. MALDI-TOF MS spectrum of products obtained after incubation of purified *Lp*MDGH1 with barley β-glucan (A) and lichenan (B) (4.75 μg enzyme per mg substrate, 50 mM ammonium acetate buffer pH 6, 320 rpm, 24 h). The reaction with enzyme and substrate (red) shows release of gluco-oligosaccharides with various degrees of polymerization (DP), while the same reaction without enzyme (negative control) only shows a minor signal for the low molecular weight oligosaccharides (blue).


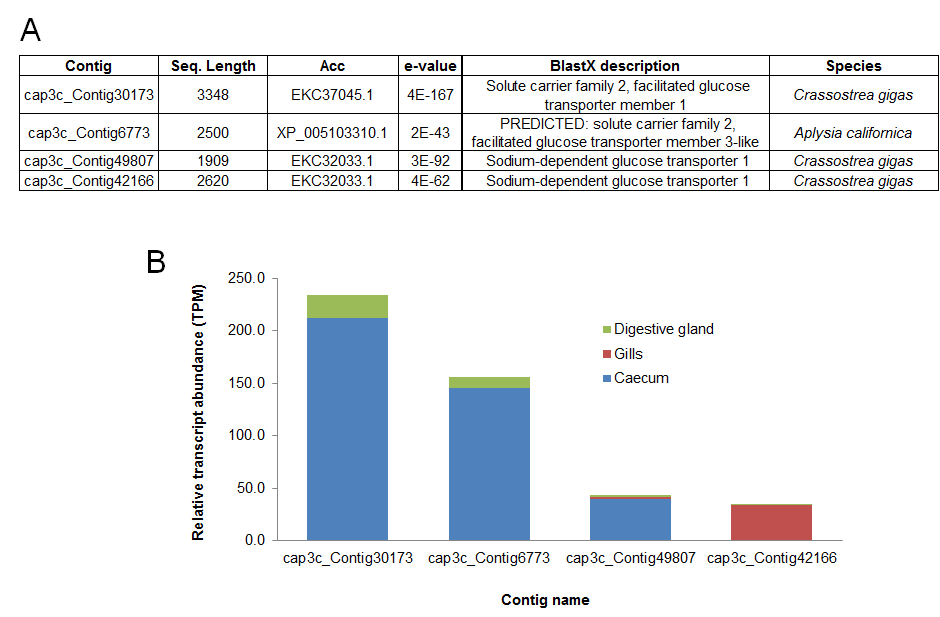


Fig. S6. Putative glucose transporters identified in the transcriptome of *L. pedicellatus*. (A) Table showing the most abundant contigs annotated as putative glucose transporters, including sequence length, identifier (Acc), e-value, description (BlastX vs NCBI nr databases) and top-hit species. (B) Relative transcript abundance (TPM = Transcripts Per kilobase Million) of the putative glucose transporters shown in (A).

Table S1. Transcriptome assembly statistics.


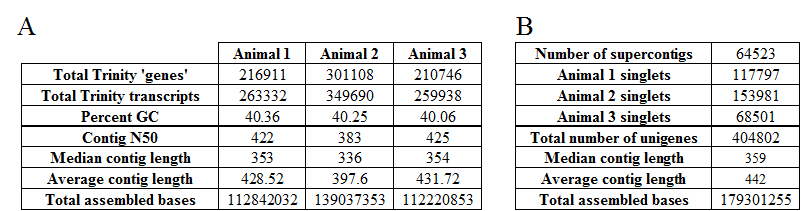


(A) Statistics of the Trinity assembly of three organs (digestive gland, cecum and gills) from three biological replicates (Animal 1/2/3) of *L. pedicellatus*. (B) Statistics of the CAP3 supercontig assembly of the contigs obtained from Trinity.

Table S2. List of endogenous CAZymes (non-bacterial) with high expression in the digestive gland of *L. pedicellatus*.

| **Contig name** | **Seq length** | **Gland TPM** | **Cecum TPM** | **Gills TPM** | **CAZyme** |
| --- | --- | --- | --- | --- | --- |
| A5_50271_c0_g5_i3 | 679 | 250.4 | 0.0 | 0.0 | GH45 |
| cap3c_Contig46568 | 1600 | 185.9 | 0.2 | 0.1 | GH9 |
| A3_73911_c3_g14_i1 | 1173 | 147.9 | 0.4 | 0.0 | GH9 |
| cap3c_Contig29275+cap3c_Contig58057 (*Lp*MDGH1) | 8905 | 106.5 | 0.1 | 0.0 | GH1 |
| cap3c_Contig22838 | 718 | 97.4 | 0.0 | 0.0 | GH45 |
| cap3c_Contig58060 | 655 | 80.0 | 0.0 | 0.0 | GH1 |
| cap3c_Contig29208 | 2407 | 74.8 | 0.2 | 0.0 | GH13 |
| cap3c_Contig46567 | 764 | 53.4 | 0.4 | 0.6 | GH9 |
| A3_80793_c1_g4_i4 | 507 | 49.6 | 21.0 | 1.6 | GH18 |
| cap3c_Contig40466 | 1726 | 40.9 | 0.0 | 0.1 | GH9 |
| cap3c_Contig28137 | 1251 | 39.4 | 0.0 | 0.1 | GH5 |
| cap3c_Contig53077 | 1148 | 37.3 | 0.7 | 0.0 | GH2 |
| cap3c_Contig52505 | 1752 | 34.9 | 0.1 | 0.0 | GH9 |
| cap3c_Contig27212 | 2210 | 32.4 | 0.0 | 0.0 | GH2 |
| cap3c_Contig53239 | 1357 | 32.0 | 15.8 | 0.1 | GH18 |
| cap3c_Contig58054 | 543 | 31.2 | 0.3 | 0.0 | GH1 |
| cap3c_Contig23302 | 866 | 28.1 | 0.7 | 0.0 | GH31 |
| cap3c_Contig27564 | 881 | 25.1 | 0.0 | 0.0 | GH45 |
| cap3c_Contig27855 | 1011 | 23.8 | 0.0 | 0.0 | GH9 |
| cap3c_Contig56706 | 1159 | 21.2 | 4.2 | 0.0 | GH1 |
| cap3c_Contig11564 | 1519 | 20.5 | 24.6 | 0.0 | GH2 |
| cap3c_Contig53507 | 2869 | 19.9 | 0.0 | 0.0 | GH9 |
| cap3c_Contig58525 | 1532 | 19.7 | 0.0 | 0.0 | GH31 |
| cap3c_Contig29064 | 1416 | 17.6 | 0.0 | 0.0 | GH10 |
| cap3c_Contig30040 | 778 | 15.7 | 10.5 | 2.1 | GH13 |
| cap3c_Contig53505 | 1147 | 15.0 | 0.0 | 0.0 | GH9 |
| cap3c_Contig58393 | 1896 | 14.6 | 18.2 | 0.1 | GH2 |
| cap3c_Contig51403 | 1161 | 13.4 | 14.1 | 0.0 | GH13 |
| cap3c_Contig56104 | 723 | 12.9 | 0.0 | 0.0 | GH1 |
| cap3c_Contig55139 | 1593 | 12.8 | 0.0 | 0.0 | GH16 |
| cap3c_Contig50895 | 2814 | 12.8 | 0.1 | 0.0 | GH10 |
| cap3c_Contig43899 | 957 | 12.6 | 0.0 | 0.0 | GH9 |
| cap3c_Contig12032 | 2280 | 12.6 | 3.0 | 1.5 | GH38 |
| cap3c_Contig58215 | 1419 | 11.9 | 0.0 | 0.0 | GH1 |
| cap3c_Contig58526 | 2197 | 11.0 | 0.0 | 0.0 | GH31 |
| cap3c_Contig56106 | 2083 | 10.3 | 0.0 | 0.0 | GH1 |
| cap3c_Contig56626 | 1435 | 9.3 | 1.0 | 0.1 | GH1 |
| cap3c_Contig53345 | 1181 | 9.2 | 0.6 | 0.0 | GH1 |
| cap3c_Contig50322 | 863 | 9.1 | 0.2 | 0.0 | GH1 |
| cap3c_Contig55555 | 3956 | 8.4 | 20.1 | 8.3 | GH13 |
| cap3c_Contig17668 | 1582 | 7.9 | 0.7 | 0.0 | GH9 |
| cap3c_Contig53880 | 1150 | 7.8 | 0.5 | 0.0 | GH1 |
| cap3c_Contig10221 | 716 | 7.5 | 0.5 | 0.7 | GH1 |
| cap3c_Contig10893 | 1083 | 7.2 | 13.8 | 7.2 | GH22 |
| cap3c_Contig62710 | 2822 | 6.3 | 3.8 | 2.3 | GH20 |
| cap3c_Contig45241 | 904 | 5.4 | 2.5 | 1.6 | GH59 |
| cap3c_Contig52415 | 963 | 4.9 | 0.0 | 0.1 | GH9 |
| cap3c_Contig45611 | 821 | 4.9 | 3.0 | 4.7 | GH18 |
| cap3c_Contig30101 | 2662 | 4.7 | 11.8 | 3.1 | GH13 |
| cap3c_Contig12084 | 3409 | 4.6 | 6.8 | 5.9 | GH84 |

In order to increase the stringency of the analysis and avoid artefactual over representation of short (partial) sequences, only contigs longer than 500 bp were considered. Relative transcript abundance was calculated based on TPM (Transcripts Per kilobase Million). Reported values equal the average of two biological replicates for all three tissues (gland, cecum and gills). The fourth most abundantly expressed CAZyme in the digestive gland corresponds to *Lp*MDGH1. Sequences were annotated using the CAZYmes Analysis Toolkit (CAT) on the BioEnergy Science Center website (<http://mothra.ornl.gov/cgi-bin/cat/cat.cgi>), setting an e-value threshold of 10^-5^.

Table S3. List of endogenous CAZymes (non-bacterial) with high expression in the cecum of *L. pedicellatus*.

| **Contig name** | **Seq length** | **Gland TPM** | **Cecum TPM** | **Gills TPM** | **CAZyme** |
| --- | --- | --- | --- | --- | --- |
| cap3c_Contig29009 | 1302 | 0.0 | 223.2 | 0.0 | GH30 |
| cap3c_Contig53922 | 1338 | 0.0 | 218.9 | 0.1 | GH30 |
| cap3c_Contig26022 | 1200 | 0.0 | 25.4 | 0.0 | GH1 |
| cap3c_Contig11564 | 1519 | 20.5 | 24.6 | 0.0 | GH2 |
| cap3c_Contig54043 | 1204 | 0.0 | 21.0 | 0.0 | GH1 |
| A3_80793_c1_g4_i4 | 507 | 49.6 | 21.0 | 1.6 | GH18 |
| cap3c_Contig55555 | 3956 | 8.4 | 20.1 | 8.3 | GH13 |
| cap3c_Contig26023 | 1922 | 0.0 | 20.1 | 0.0 | GH1 |
| cap3c_Contig58393 | 1896 | 14.6 | 18.2 | 0.1 | GH2 |
| cap3c_Contig53596 | 1863 | 0.2 | 16.9 | 0.1 | GH1 |
| cap3c_Contig9408 | 1589 | 0.2 | 16.1 | 0.0 | GH1 |
| cap3c_Contig53239 | 1357 | 32.0 | 15.8 | 0.1 | GH18 |
| cap3c_Contig35107 | 4762 | 2.4 | 15.7 | 1.7 | GH20 |
| cap3c_Contig10609 | 2516 | 0.0 | 15.4 | 0.1 | GH1 |
| cap3c_Contig28811 | 1755 | 0.3 | 14.2 | 0.0 | GH35 |
| cap3c_Contig51403 | 1161 | 13.4 | 14.1 | 0.0 | GH13 |
| cap3c_Contig51669 | 2650 | 0.0 | 14.0 | 0.0 | GH1 |
| cap3c_Contig10893 | 1083 | 7.2 | 13.8 | 7.2 | GH22 |
| cap3c_Contig58727 | 2881 | 0.1 | 12.7 | 0.0 | GH1 |
| cap3c_Contig30101 | 2662 | 4.7 | 11.8 | 3.1 | GH13 |
| cap3c_Contig53037 | 2258 | 0.0 | 10.7 | 0.0 | GH1 |
| cap3c_Contig30040 | 778 | 15.7 | 10.5 | 2.1 | GH13 |
| cap3c_Contig24536 | 2564 | 1.3 | 8.5 | 1.1 | GH20 |
| A2_54531_c0_g1_i2 | 1296 | 0.0 | 7.4 | 0.3 | GH1 |
| cap3c_Contig12084 | 3409 | 4.6 | 6.8 | 5.9 | GH84 |
| cap3c_Contig54018 | 1324 | 4.4 | 6.5 | 3.3 | GH2 |
| cap3c_Contig23232 | 1533 | 2.2 | 4.5 | 0.8 | GH23 |
| cap3c_Contig56706 | 1159 | 21.2 | 4.2 | 0.0 | GH1 |
| cap3c_Contig62710 | 2822 | 6.3 | 3.8 | 2.3 | GH20 |
| cap3c_Contig45611 | 821 | 4.9 | 3.0 | 4.7 | GH18 |
| cap3c_Contig51811 | 1594 | 2.9 | 3.0 | 2.1 | GH9 |
| cap3c_Contig12032 | 2280 | 12.6 | 3.0 | 1.5 | GH38 |
| cap3c_Contig29209 | 1349 | 3.7 | 3.0 | 0.8 | GH13 |
| cap3c_Contig47433 | 1130 | 2.6 | 2.5 | 2.0 | GH29 |
| cap3c_Contig47200 | 1572 | 3.4 | 2.5 | 3.0 | GH18 |
| cap3c_Contig54162 | 1011 | 3.4 | 2.5 | 0.0 | GH13 |
| cap3c_Contig45241 | 904 | 5.4 | 2.5 | 1.6 | GH59 |
| cap3c_Contig3041 | 1590 | 3.1 | 2.1 | 3.0 | GH1 |
| cap3c_Contig24624 | 2103 | 4.2 | 2.0 | 0.0 | GH13 |
| cap3c_Contig54553 | 3252 | 1.4 | 1.9 | 1.8 | GH31 |
| cap3c_Contig9406 | 2378 | 2.4 | 1.9 | 2.4 | GH20 |
| cap3c_Contig6229 | 1630 | 1.8 | 1.8 | 0.4 | GH5 |
| cap3c_Contig59941 | 1436 | 0.6 | 1.6 | 2.1 | GH38 |
| cap3c_Contig7518 | 1607 | 0.4 | 1.3 | 1.2 | GH79 |
| cap3c_Contig11986 | 2375 | 3.6 | 1.2 | 1.2 | GH27 |
| cap3c_Contig23861 | 893 | 2.6 | 1.0 | 0.4 | GH3 |

In order to increase the stringency of the analysis and avoid artefactual over representation of short (partial) sequences, only contigs longer than 500 bp were considered. Relative transcript abundance was calculated based on TPM (Transcripts Per kilobase Million). Reported values equal the average of two biological replicates for all three tissues (gland, cecum and gills). Sequences were annotated using the CAZYmes Analysis Toolkit (CAT) on the BioEnergy Science Center website (<http://mothra.ornl.gov/cgi-bin/cat/cat.cgi>), setting an e-value threshold of 10^-5^.

Table S4. List of bacterial CAZymes with high expression in the gills of *L. pedicellatus*.

| **Contig name** | **Seq length** | **Gland TPM** | **Cecum TPM** | **Gills TPM** | **CAZyme** |
| --- | --- | --- | --- | --- | --- |
| cap3c_Contig22444 | 543 | 0.0 | 0.0 | 508.9 | GH6 |
| A3_79180_c3_g64_i1 | 537 | 0.0 | 0.0 | 223.3 | GH11 |
| cap3c_Contig26869 | 1257 | 0.0 | 0.4 | 188.6 | AA10 |
| cap3c_Contig26128 | 1528 | 0.0 | 0.0 | 181.6 | GH5 |
| cap3c_Contig26727 | 1568 | 0.1 | 0.0 | 179.5 | GH11 |
| cap3c_Contig26870 | 510 | 0.0 | 0.0 | 121.1 | AA10 |
| cap3c_Contig54834 | 1983 | 0.1 | 0.1 | 59.1 | GH6 |
| cap3c_Contig26127 | 1936 | 0.1 | 0.0 | 54.7 | GH5 |
| cap3c_Contig27252 | 1892 | 0.0 | 0.1 | 38.8 | GH10 |
| cap3c_Contig18663 | 748 | 0.0 | 0.3 | 37.9 | CE3 |
| cap3c_Contig55597 | 2442 | 0.2 | 0.3 | 36.1 | GH5 |
| cap3c_Contig11902 | 835 | 0.0 | 0.0 | 35.3 | CE15 |
| cap3c_Contig22006 | 736 | 0.0 | 0.0 | 35.1 | GH5 |
| cap3c_Contig25612 | 1574 | 0.2 | 0.2 | 33.9 | GH5 |
| cap3c_Contig49865 | 1541 | 0.0 | 0.0 | 31.0 | AA10 |
| cap3c_Contig27359 | 1416 | 0.0 | 0.0 | 30.2 | CE3 |
| cap3c_Contig27302 | 1053 | 0.0 | 0.0 | 25.1 | CE4 |
| cap3c_Contig49707 | 833 | 0.0 | 0.0 | 24.7 | GH11 |
| cap3c_Contig24190 | 608 | 0.4 | 0.0 | 19.5 | GH115 |
| cap3c_Contig51309 | 2461 | 0.2 | 0.2 | 18.2 | GH5 |
| cap3c_Contig26574 | 1412 | 0.0 | 0.3 | 15.9 | GH9 |
| cap3c_Contig23079 | 803 | 0.0 | 0.3 | 13.2 | GH9 |
| cap3c_Contig49039 | 879 | 0.0 | 0.0 | 12.1 | GH5 |
| cap3c_Contig11945 | 2008 | 0.2 | 0.0 | 11.5 | GH10 |
| cap3c_Contig35608 | 1744 | 0.2 | 0.0 | 10.5 | GH10 |
| cap3c_Contig23700 | 1223 | 0.1 | 0.3 | 10.4 | PL3 |
| cap3c_Contig29672 | 1094 | 0.0 | 0.0 | 9.8 | GH5 |
| cap3c_Contig41761 | 1561 | 0.0 | 0.0 | 9.2 | GH3 |
| cap3c_Contig27361 | 1630 | 0.0 | 0.1 | 8.4 | GH11 |
| cap3c_Contig31373 | 1657 | 0.0 | 0.0 | 8.2 | CE15 |
| cap3c_Contig26768 | 1468 | 0.0 | 0.0 | 8.1 | AA10 |
| cap3c_Contig30157 | 3322 | 0.0 | 0.0 | 8.0 | GH16 |
| cap3c_Contig26689 | 1515 | 0.0 | 0.0 | 7.1 | GH10 |
| cap3c_Contig45784 | 1562 | 0.0 | 0.0 | 6.9 | GH10 |
| cap3c_Contig51253 | 2016 | 0.3 | 0.4 | 6.9 | GH2 |
| cap3c_Contig27551 | 1204 | 0.0 | 0.0 | 6.7 | GH45 |
| cap3c_Contig27627 | 2800 | 0.0 | 0.2 | 6.2 | GH105 |
| cap3c_Contig34672 | 1821 | 0.0 | 0.0 | 6.1 | GH5+GH6 |
| cap3c_Contig29580 | 2219 | 0.0 | 0.1 | 5.6 | GH115 |
| cap3c_Contig31013 | 1973 | 0.0 | 0.0 | 5.6 | GH3 |
| cap3c_Contig3235 | 1510 | 0.0 | 0.0 | 5.2 | GH10 |
| cap3c_Contig26831 | 1175 | 0.0 | 0.0 | 5.2 | CE3 |
| cap3c_Contig4957 | 1132 | 0.0 | 0.0 | 4.6 | GH62 |
| cap3c_Contig59310 | 1786 | 0.0 | 0.0 | 4.4 | GH5 |
| cap3c_Contig18389 | 1631 | 0.1 | 0.2 | 4.4 | GH5 |
| cap3c_Contig41324 | 1580 | 0.0 | 0.1 | 2.6 | GH8 |
| cap3c_Contig27629 | 2134 | 0.0 | 0.0 | 2.3 | GH105 |

In order to increase the stringency of the analysis and avoid artefactual over representation of short (partial) sequences, only contigs longer than 500 bp were considered. Relative transcript abundance was calculated based on TPM (Transcripts Per kilobase Million). Reported values equal the average of two biological replicates for all three tissues (gland, cecum and gills). Sequences were annotated using the CAZYmes Analysis Toolkit (CAT) on the BioEnergy Science Center website (<http://mothra.ornl.gov/cgi-bin/cat/cat.cgi>), setting an e-value threshold of 10^-5^.

Additional text

Protein sequence of *Lp*MDGH1

MWWVCAVLCLTAFTEAQFGFGPGSQFLFGSFPDDFQWGLTNTELNYDGAIDYIPAEIAAIKALGVSSYRVEIDWLFNHSVNLAVNIEYYTELAKELEEIGVELKITLFNTRYPYGRFGWDGCAAAAEVIEALGDKVAAWTTFSNPRYIFQPASVTIDVHNCVYDTIKNASLTAPVGIDLSAGWYAPARFHERDFNASGRAFFNDIPAYMEGLKTDFLGLIYNGAFTITHDDTEGNPYSYRINTSPFWERDGWSYFPEGLRGVLFYVSAAYPDVDVYVQNLGRPTDENEITDDQRVEYLRY

HIDEVLKSVTQDNSTVVGLSVFSLVDSEADPNFHSGLYQNLSPPRPKASAILLTQLIKDNGFEPGYNGIGGFPSGPVEFEDEFYYDSFPDDFAWSTATSAYQIEGGWDEDGKGESIWDTHAHTPGNIYMNATGDVADDSYHKYMEDIKALVALETKYYRFSLAWTRLMPDGTKASINQAGIDHYSKVIDALLDAEITPMITLFHWDLPQALQDNGGWLNESTIGHFRDYAELCYTHFGDRVKFWITFNEPKITTLHSYGGATFPPATQDWQAVYTSAHNLIKAHAEAYHAYNKSQGGQVG

ITVAAGWGEPFNMYNPSDLAASDRSMAFEVGWFIHPIYINGDYPEVMKYQVAMKSRQQGYNVSRLPEFTDEEKARINGTYDFYGLNFYTASMVSTDPFSFPDRTGNYYNDMDVSSWKDPTWLGSGSDWLKVTPFGIRKALNYIKYNYKNVPVYITENGVSDRNGTLRDYHRIHFYRSYINEVLKAIKLDGCNVKGYTAWSLMDNFEWNTGFRERFGLYYVNFSDPELERVPKASAYWYTQLVEENAFSHGYPGLGGRGVSPDYVDKMYYNTFDDDFEWGVGTEAYEVEGGWKADGKGWSI

LDTWAQAGNFPEGQTGNKAADSYHQYMEDVKIIGDLGVDFYQFSISWSRIFPTGVEPVNQAGVDYYMSLIKALKSSGIKPVAQLYASDLPQDLEDLGGWLNDSSIQWFEDYAQFCFETFGEDVERWITFNEPALTSNLGYGAGFFAPGQNNSGINDYIVGYHMLKSHAAAHKVFKSTNATAGGSIGIFLTGTWNEPFDEFSPADWMASERAMQFSLGWFARPIFGDGQYPWVMKEAAGDRLPDLTDEDMAEIKGSADFLGLTVFSSSLCSHLNESAFDFTFEGFFADQATFCFPDFNQTT

SPARKTLNWIKNTYGDINVYVRSGVSSNFADIGEHHITLVNEVLKANKLDGVNVKGYTAFSLVDGFEMAFQGLNGRYGLYNVDFDSEGFERTPKMAAVNYSHIVQVNGFKEVDFDVYIPMEHEMLEGKFPDDFIIGTASASYQIEGAWNESGKGPSIWDTFAHEGGHIWQNDSGDVAADGYHHYLEDIRLIKSLNVPLYRLSIAWTRIYPKGYGELNQAGVDFYNNFIDALLEIGVEPLVTLYHWDLPQGLQDFGGWLNESTTDAFENYADTCFRLFGDRVKRWLTLNEPWVCSKQGFED

GSYAPGHVTSGTEIYQAGHNMLLAHAKGFRVYDEKYRTTQHGRVGITLNCDWWIPRNPDDPADWIAAERAMAFHCGWFKNPIFKNGDYPEVMKDYVARYSEAQGLSKSRLPEFTDEQKQMLAGSADFLGLNHYTTNRAKYRVCGENDTGYYCDMEVESDQDPSWPGSGSVWLKVNPPGFRRLLNYIKDNYNNVPVFVTENGVTDNNGTVNDGHRISYYNKYMNELLKAVVLDGCNIEVYVAWAIVDFFEWSSGFHEKFGFFHVDLNDPARPRSSKESAIWFRDMVMYGYTNKTLPMADEF

YYGRFPNGFIWAVATASYQIEGAWDEDGKGPNIWDTFTHEGGNIKQNDTGDIATDSYHKYPEDINLLRALGVSHYRFSLSWARIMPMGYGEVNQAGIDFYNDFIDMLIAGGIEPMITLYHWDLPQAIQDEYGGWPNRSVADLFEAYADVCFEAFGDRVKFWITLNEPWCTAFLGYGNGQDAPGIVDSGISDYQAAHTLILSHARAYRLYERKYKRYQKGRCGITLNSDWASPKNASDVKDWAAAERRVQFHLGWFANPIYVNGQYPPIMQFQIHRKSDMQGLNETRLPQFTPFESFLVQG

SSDFFGLNSYTTVRSADAEWGADGNYYNDMDVAGDADPSWPASGSSWLYVVPWGIRHLLNFIKDNYGDPEIYITENGVSDNNGTLEDDHRVEFYRSYTNEVLKAINLDDVNVKGYTAWSLLDNFEWSRGYTEYFGMHSVNMSDPERPRAAKKSAEFYRELILRNGFSADPFPTFPIQMEDEFLYGTFPDNFAWSVATAAYQVEGAWDEDGKGPSVWDTFTHAGGKVKNNDTGDVACDSYHKTAEDVQLLVDLKVSHYRFSIAWTRIMPDGTNSTINEPGIAYYNDLIDSLLAANITPMVT

MNHWDLPQGLEDMDGWVNASIAEYFRDYADVLYETFGDRVKYWITHNEPWVIALHGYETAEKAPGRVNHGYEAAHNLLRAHALAYKLYNDKYRDTQGGMVGITLNHSPGLPLDPDSISDIIATEVYNQFGLGWFAQPVFGNGDYPDIMKWQVGNKSLEQGYPESRLPAFTDEEKDMLSGSSDFFGYNPYTAVKVTDKVSPLTQVGYYPDQDVAQSQDPSWGDSGSFWLKVVPTAMRTGLNFIRFRYNNYPVYITENGVSDHNGALNDTWRIEYYKGYINEMLKAIVLDGCDVKGYTAWSF

MDNFEWQEGYSERFGLHYVDFEDPDRPRTPKESAKYYTSIIEANGFPADESA
